# Use of Overlapping Group LASSO Sparse Deep Belief Network to Discriminate Parkinson's Disease and Normal Control

**DOI:** 10.3389/fnins.2019.00396

**Published:** 2019-04-29

**Authors:** Ting Shen, Jiehui Jiang, Wei Lin, Jingjie Ge, Ping Wu, Yongjin Zhou, Chuantao Zuo, Jian Wang, Zhuangzhi Yan, Kuangyu Shi

**Affiliations:** ^1^Shanghai Institute for Advanced Communication and Data Science, Shanghai University, Shanghai, China; ^2^Key laboratory of Specialty Fiber Optics and Optical Access Networks, Joint International Research Laboratory of Specialty Fiber Optics and Advanced Communication, Shanghai University, Shanghai, China; ^3^Department of Neurosurgery, 904 Hospital of PLA, Anhui Medical University, Wuxi, China; ^4^PET Center, Huashan Hospital, Fudan University, Shanghai, China; ^5^Institute of Functional and Molecular Medical Imaging, Fudan University, Shanghai, China; ^6^Human Phenome Institute, Fudan University, Shanghai, China; ^7^Department of neurology, Huashan Hospital, Fudan University, Shanghai, China; ^8^Department of Nuclear Medicine, University Hospital Bern, Bern, Switzerland; ^9^Department of Nuclear Medicine, Technische Universitat Munchen, Munich, Germany

**Keywords:** Parkinson's disease, Deep Belief Network, overlapping group LASSO, sparse representation, deep learning, early diagnose

## Abstract

As a medical imaging technology which can show the metabolism of the brain, 18F-fluorodeoxyglucose (FDG)-positron emission tomography (PET) is of great value for the diagnosis of Parkinson's Disease (PD). With the development of pattern recognition technology, analysis of brain images using deep learning are becoming more and more popular. However, existing computer-aided-diagnosis technologies often over fit and have poor generalizability. Therefore, we aimed to improve a framework based on Group Lasso Sparse Deep Belief Network (GLS-DBN) for discriminating PD and normal control (NC) subjects based on FDG-PET imaging. In this study, 225 NC and 125 PD cohorts from Huashan and Wuxi 904 hospitals were selected. They were divided into the training & validation dataset and 2 test datasets. First, in the training & validation set, subjects were randomly partitioned 80:20, with multiple training iterations for the deep learning model. Next, Locally Linear Embedding was used as a dimension reduction algorithm. Then, GLS-DBN was used for feature learning and classification. Different sparse DBN models were used to compare datasets to evaluate the effectiveness of our framework. Accuracy, sensitivity, and specificity were examined to validate the results. Output variables of the network were also correlated with longitudinal changes of rating scales about movement disorders (UPDRS, H&Y). As a result, accuracy of prediction (90% in Test 1, 86% in Test 2) for classification of PD and NC patients outperformed conventional approaches. Output scores of the network were strongly correlated with UPDRS and H&Y (*R* = 0.705, *p* < 0.001; *R* = 0.697, *p* < 0.001 in Test 1; *R* = 0.592, *p* = 0.0018, *R* = 0.528, *p* = 0.0067 in Test 2). These results show the GLS-DBN is feasible method for early diagnosis of PD.

## Introduction

Parkinson's disease (PD) is a long-term degenerative disease of the central nervous system which effects 2–3% of the world's population over 65 years old, and its incidence is increasing in recent years(Postuma and Berg, 2017). Accurate early diagnosis of PD is crucial for treatment and prognosis.

Imaging disease-specific patterns of regional glucose metabolism with 18F-fluorodeoxyglucose (FDG)-positron emission tomography (PET) allows for accurate diagnosis of PD, this has been increasingly acknowledged in recent years (Eckert et al., 2005; Dabrowska et al., 2015; Meyer et al., 2017; Politis et al., 2017). Some studies (Juh et al., 2004; Brajkovic et al., 2017) used voxel-based statistical analyses or network analysis in comparison to normal control (NC). For example, Juh et al. used statistical parametric mapping to determine useful metabolic patterns in diagnosing PD (Juh et al., 2004). Brajkovic et al. combined visual assessment of individual scans with statistical parametric mapping (Brajkovic et al., 2017). These researches showed that compared with NC, glucose metabolism of PD patients in sensorimotor cortex, lateral frontal and parietooccipital areas was decreased (Meles et al., 2017), which is of great value for the early diagnose of PD.

Currently, with the development of artificial intelligence and data-driven analysis, various computer-aided-diagnosis systems based on machine learning or deep learning(Chandra and Sharma, 2016; Chen et al., 2017) methods have been developed to identify brain disease related alterations in neuroimaging datasets. For instance, some studies (Tang et al., 2010; Garraux et al., 2013; Tripathi et al., 2015) classify PD patients based on automated statistical analysis. Garraux et al. applied logistic regression based on the expression of metabolic covariance patterns. Tripathi et al. used a relevance vector machine in combination with bootstrap resampling for multiclass classification. Also, in Matthews's research (Matthews et al., 2018), two machine learning approaches (Canonical Variates Analysis and Scaled Subprofile Model) were used to represent the difference in motor symptoms between NC and PD patients. Methods using deep learning have also been explored to extract latent features from PET images. For example, Liu et al. extracted potential features from 83 regions of interest in magnetic resonance imaging and PET scans and trained a multilayer neural network of multiple auto-encoders to combine multimodal features for classification (Siqi et al., 2015). Suk et al. presented that a stacked auto-encoder can be used to learn the underlying non-linear complicated patterns in low-level features, for example, the relationship between features (Suk et al., 2015). Also, in Brosch et al.'s study, in order to find the modes of variation between disease parameters and demography, they proposed a low-dimensional manifold of brain volumes based DBN model (Brosch et al., 2014).

While previous studies have claimed that existing machine learning and deep learning methods achieved an acceptable classification accuracy to discriminate PD and normal controls (NC), these methods are still hampered by over-fitting and poor generalizability, due to few labeled samples in neuroimaging datasets. To solve above problems, scholars have used deep learning models with multi-parameters, e.g., deep belief network (DBN), to avoid models' poor generalizability from traditional machine learning methods (Yoshida and Miyato, 2017; Xu et al., 2018). In addition, they have also proposed to add regular terms to the objective function, which could optimize the loss function, reduce the complexity of deep learning models, and prevent models' over-fitting (Mei et al., 2015). For these reasons, considering the feature distribution of PET images, in this paper, based on DBN, we add the Group Lasso Sparse (GLS) model as a regular term to the objective function to prevent the model over-fitting, and at the same time, to learn the multi-level imaging features such as texture or edge information for classifying PD vs. NC. To evaluate the effectiveness of our method, we also compared our model with other deep learning models.

## Group Lasso Sparse Deep Belief Network (GLS-DBN) Algorithm

In this paper, we propose an improved DBN method, Group Lasso Sparse Deep Belief Network (GLS-DBN), for feature learning and classification of PET images. As a deep architecture, DBN is suitable to deliver non-linear and complicated machine learning information (Liu et al., 2011; Rui and Yang, 2017; Zheng and Lu, 2017; Prasetio et al., 2018). Sparsity has become a key ingredient for improving DBN because compared with non-sparse representations, sparse representations are more efficient from the point of view of information theory, which allow the change of the effective number of bits per example in a fixed-size representation (Ranzato et al., 2007; Luo et al., 2010; Halkias et al., 2013). Sparsity is generally introduced into DBN by adding a sparse penalty to the objective function and considering it as a convex optimization problem. For example, based on the DBNs of Hinton et al. (2006), Lee et al. proposed a sparse DBN which faithfully simulating some properties of visual region V2 (Lee et al., 2007). Ji et al. proposed a sparse-response DBN based on rate distortion theory, in which the distortion function was based on Kullback-Leibler divergence between equilibrium distribution in DBN model and data distribution, then a small code rate was realized by adding sparse response regularization (Ji et al., 2014). Xu et al. examined the problem of invariance existing in sparse regular term and proposed an improved sparse DBN (Xu et al., 2018). This model uses Laplace distribution to induce the sparse state of hidden layer nodes, and uses location parameters in the distribution to control the intensity of sparsity. In addition to these, Keyvanrad et al added normal regularization term in DBN which make the whole model has different response according to difference between hidden units' activation and fixed value (Keyvanrad and Homayounpour, 2017). Compared with base DBN model (without sparse penalty), all of these models achieved better performance in natural image recognition, but it is unknown whether these methods can be applied to PET images. Therefore, to evaluate PET image patterns, we combined traditional DBN with the overlapping group lasso model and propose a novel sparse DBN model(Rao et al., 2015; Jian et al., 2017; Liu et al., 2017; Yuan et al., 2018), GLS-DBN, adding a sparse penalty to learn useful low-level feature representations.

GLS-DBN is based on GLS Restricted Boltzmann Machine (GLS-RBM). As an improved Restricted Boltzmann Machine (RBM) (Fischer and Igel, 2012), GLS-RBM combines the overlapping group lasso model with the pre-training of traditional RBM, grouping its hidden units according to the same overlap ration of each group. Through this, GLS-RBM connects similar features between groups. When a large number of similar features exist discretely in multiple groups, multiple groups are activated simultaneously, effectively solving the problem of over fitting in the traditional learning model and improving the recognition rate of the model.

We implement the GLS-RBM model by adding a sparse penalty to the objective function. In this paper, we also use the Cauchy distribution to replace the traditional L1 normal form between groups in the overlapping groups Lasso model, making the entire model sparser at the group level (Lü et al., 2016). For sample collection: {*v*^1^, *v*^2^ … *v*^*m*^}, the optimization model of unsupervised pre-training of GLS-RBM is:

(1) $$\begin{array}{l} F=\;F_{unsup}+\;\tau F_{sprase} \end{array}$$

(2) $$\begin{array}{l} F_{sprase}=\lambda F_{Lasso}+\;\varphi F_{Cachy} \end{array}$$

Where *F*_*unsup*_ is the likelihood function of the RBM. The new objective function of the optimization GLS-RBM model after adding a sparse penalty is:

(3) $$\begin{array}{l} \begin{array}{l} {\text{minimize}}_{\left\{w_{ij},b_{i},c_{j}\right\}}F=\;-\frac{1}{m}\sum_{l=1}^{m}log\sum_{h}^{\;}P\left(v^{\left(l\right)},h^{\left(l\right)}\right)\;\\ \;+\;\tau \;\sum_{l=1}^{m}F_{sprase} \end{array} \end{array}$$

**Figure 1:**
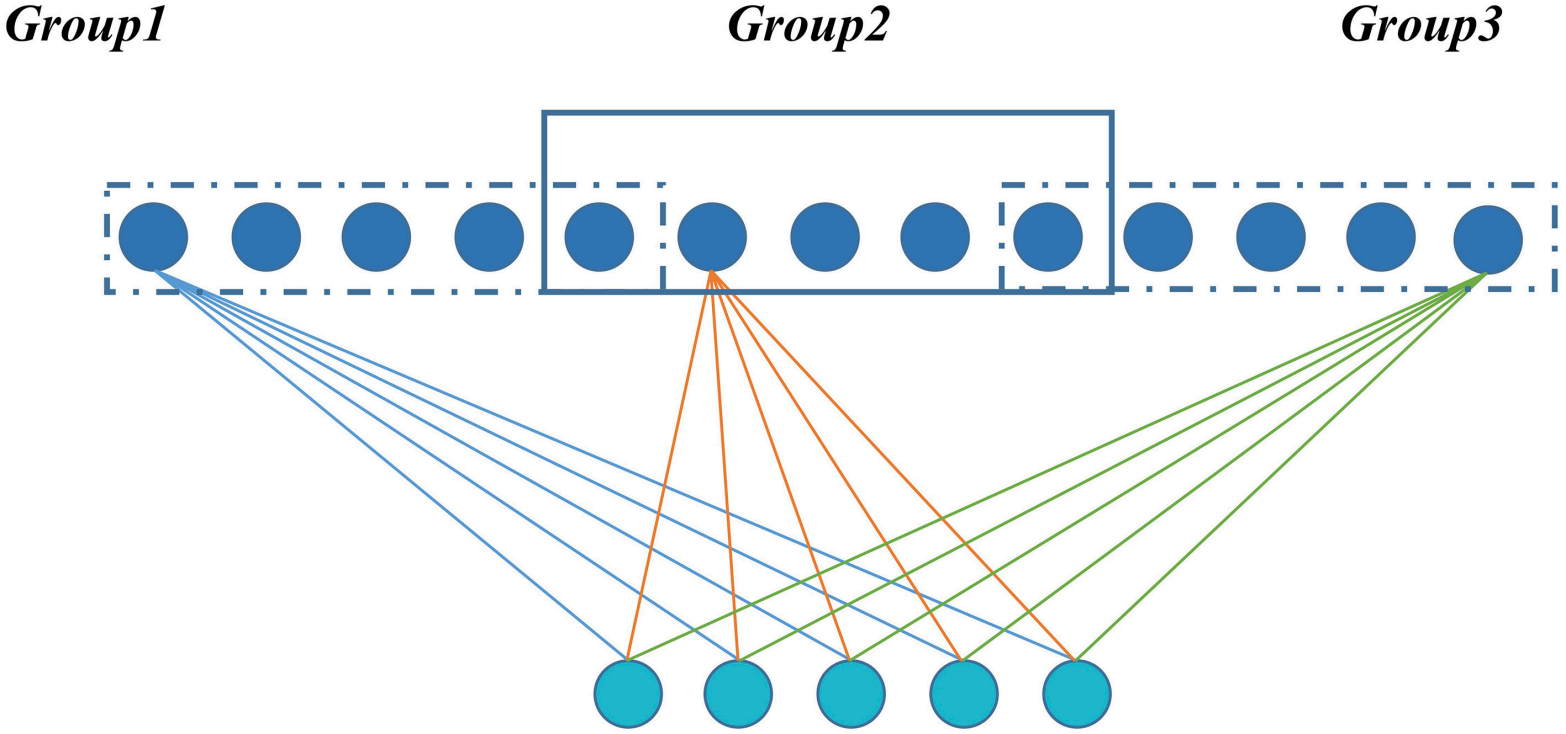
A simple RBM model based overlapping Group Lasso model. All visible units are evenly divided into three groups, and overlapping rate is 0.2.

For a GLS-RBM model, all hidden units {*h*_1_, *h*_2_ … *h*_*n*_} are evenly distributed to I overlapping groups. Each group has the same number of nodes, and there is overlap between each group. The degree of overlapping is determined by α (between-group and group). Figure 1 shows a simple GLS-RBM model based overlapping group lasso.

The *F*_*sprase*_ in the whole layer becomes:

(4) $$\begin{array}{l} \begin{array}{lll} F_{sprase} & = & \text{λ}F_{Lasso}+\;\varphi F_{Cachy}\\ & = & \lambda \sum_{i=1}^{I}\sqrt{\sum_{n\in Group_{i}}^{\;}p{\left(h_{n}=1\;|\;v\right)}^{2}}\;+\;\varphi \sum_{j=1}^{n}L\left(\gamma ,\mu ,p_{j}\right)\; \end{array} \end{array}$$

(5) $$\begin{array}{lll} \text{L}\left(\gamma ,\mu ,p\right) & = & \;\frac{1}{\pi }\frac{\gamma }{{\left(x-\mu \right)}^{2}+\;\gamma^{2}}=\;\frac{1}{\pi \gamma }\frac{1}{\left[1+\;{\left(\frac{p_{j}-\mu }{\gamma }\right)}^{2}\right]} \end{array}$$

Where *p* is the probability distribution of the hidden unit, γ is the scale parameter, which controls the degree of sparsity, and μ is the location parameter.

The Gradient descent algorithm is used to iteratively solve and update parameters of the function. We use the objective function with penalty to update the weight *w*, and the hidden layer bias *b*. The visible layer bias is obtained according to the original objective function. The gradient of the objective function is solved as follows:

(6) $$\begin{array}{lll} \frac{\partial F}{\partial W_{ij}} & = & \;\frac{\partial }{\partial W_{ij}}\left(\frac{1}{m}\overset{m}{\underset{l=1}{\sum }}ln\left(P\left(v^{\left(l\right)}\right)\right)+\;\frac{\partial F_{sprase}}{\partial W_{ij}}\right) \end{array}$$

(7) $$\begin{array}{lll} \frac{\partial F}{\partial b_{j}} & = & \;\frac{\partial }{\partial b_{j}}\left(\frac{1}{m}\overset{m}{\underset{l=1}{\sum }}ln\left(P\left(v^{\left(l\right)}\right)\right)+\;\frac{\partial F_{sprase}}{\partial b_{j}}\right) \end{array}$$

(8) $$\begin{array}{l} \frac{\partial F}{\partial a_{j}}=\frac{\partial }{\partial a_{j}}\left(\frac{1}{\text{m}}\overset{m}{\underset{l=1}{\sum }}ln\left(P\left(v^{\left(l\right)}\right)\right)\right) \end{array}$$

Because

(9) $$\begin{array}{l} \frac{\partial F_{sprase}}{\partial p_{j}}=\frac{1}{2}\sum_{i=1}^{I}\frac{2\times p_{j}^{\left(l\right)}}{\sqrt{\sum_{j\in Group_{i}}p_{j}{}^{{\left(l\right)}^{2}}}}\\ +\frac{1}{2m}\sum_{l=1}^{m}\sum_{i=1}^{I}\frac{2\times p_{j}^{\left(l\right)}}{\sqrt{\sum_{j\in Group_{i}}p_{j}^{{\left(l\right)}^{2}}}}-\frac{1}{\pi \gamma^{2}}\\ \times \frac{1}{m}\sum_{l=1}^{m}\frac{2\left(\frac{p_{j}^{\left(l\right)}-\mu }{\gamma }\right)}{{\left(1+{\left(\frac{p_{j}^{\left(l\right)}-\mu }{\gamma }\right)}^{2}\right)}^{2}} \end{array}$$

The second item with sparse penalty is expanded as follows:

(10) $$\begin{array}{l} \frac{\partial F_{sprase}}{\partial W_{ij}}=\;\overset{n}{\underset{j=1}{\sum }}\frac{\partial F_{sprase}}{\partial p_{j}}\;\times \;\frac{1}{m}\overset{m}{\underset{l=1}{\sum }}p_{j}^{l}\left(1-p_{j}^{l}\right)v_{i}^{l} \end{array}$$

(11) $$\begin{array}{l} \frac{\partial F_{sprase}}{\partial b_{j}}=\;\overset{n}{\underset{j=1}{\sum }}\frac{\partial F_{sprase}}{\partial p_{j}}\;\times \;\frac{1}{m}\overset{m}{\underset{l=1}{\sum }}p_{j}^{l}\left(1-p_{j}^{l}\right) \end{array}$$

In whole GLS-DBN, multiple basic GLS-RBMs can be stacked upon each other to form a deep hierarchy. The output of each GLS-RBM serves as the input of the next basic GLS-RBM at successive levels. In the last layer of GLS-DBN, a back propagation (BP) network is set, receiving the output feature vector of GLS-RBM as learned features, and adopting a gradient descent algorithm to fine-tune the weight of the whole network, thereby coordinating and optimizing the parameters of the whole DBN. The feature vector mapping of GLS-DBN is optimized and the size of the input space is simplified.

## Materials and Methods

### Materials

Two different cohorts of PD and NC subjects was included in this study. The first cohort came from Huashan Hospital, Fudan University, Shanghai, China. Subjects were recruited from Chinese populations and totaled 300 participants: 200 NC and 100 PD patients. Before the study, follow-up data for at least 1 year from all participants was collected. Then clinicians who were unaware of the imaging results were followed up to further confirm the clinical diagnosis. Healthy controls in this study were accepted by senior movement disorders expert neurological examination, to rule out a history of psychiatric or neurologic disorders. All participants had no drug use or exposed to antipsychotic drugs.

The subjects in the Huashan cohort were randomly divided into training & validation and test (Test 1) datasets. The Test 1 dataset consisted of 50 subjects, including 25 PD patients and 25 NC subjects. Due to the variability of sampling when grouping data sets, the random cross validation method is used in the training & validation dataset, which included 75 PD patients and 175 NC subjects randomly partitioned into deep learning model training (80%) and validation (20%), with 50 iterations. The purpose of multiple cross-validation was to find the optimal parameter combination. Finally, the Test 1 dataset was used to verify the performance of the trained model.

The second cohort was from 904 Hospital in Wuxi, China, and included 25 NC and 25 PD patients, enrolled between 2011 and 2015. Also, before the study, necessary screening and clinical examinations from the two senior investigators of movement disorders were used to select eligible subjects. All subjects in the Wuxi cohort were used as a test dataset (Test 2) to verify the reliability and robustness of the deep learning model.

The demographic information and clinical data of two cohorts are shown in Table 1. The clinical characteristics (HY, UPDRS) were not significantly different among Training & Validation dataset and test datasets (Test 1 and Test 2) for PD or NC (*P* > 0.05). The study was ethically approved by the Institutional Review Boards at North Shore University Hospital and Huashan Hospital. The study was conducted in accordance with the Code of Ethics of the World Medical Association (Declaration of Helsinki) and the standards set by the local Institutional Review board and funding agencies. After a detailed explanation of the scanning procedure, each subject received written consent from each institution. During or after data collection, authors can access information that could identify all participants.

**Table 1:** Demographic and clinical information of Huashan hospital control and Wuxi 904 hospital cohort.

	**Cohort**		***N***	**Gender(M/F)**	**Age(years)**	**H&Y**	**UPDRS**
Huashan Hospital Cohort	Training & Validation dataset	NC	175	98/77	49.5 (29)	N/A	N/A
		PD	75	51/24	56.5 (14.5)	2 (1)	20 (19.05)
		*P*-Value	–	0.9217^a^	0.593^b^	–	–
	Test dataset (Test 1)	NC	25	10/15	50 (22.5)	N/A	N/A
		PD	25	14/11	55 (16)	2.5 (1.5)	25 (19.5)
		*P*-Value	–	0.4218^a^	0.258^b^	–	–
Wuxi 904 Hospital Cohort	Test dataset (Test 2)	NC	25	12/13	59 (9)	N/A	N/A
		PD	25	19/6	65 (11.5)	2.5 (1.5)	28 (18.5)
		P-Value	–	0.1884^a^	0.771^b^	–	–

*Age and clinical ratings are given as. Median (Interquartile range). H & Y, Hoehn and Yahr scale; UPDRS, Unified Parkinson's Disease Rating Scale; P^a^, The chi-square test; P^b^, The two-sample t-test*.

### PET Imaging Acquisition

All participants were asked to fast before imaging. And the whole experiment was carried out in a dimly-lit room. The equipment used in this study was Siemens Biograph 64 PET/ computed tomography (CT; Siemens, Germany). After 45 min of intravenous injection of 185 MBq of FDG, the scans were performed for about 10 min. Hanning filter is used for image reconstruction and then projection, with an axial and transaxial cut-off frequency of 0.5.

### PET Pre-processing

The pre-processing of original PET data was completed by SPM5 software (Wellcome Department of Imaging Neuroscience, Institute of Neurology, London, UK). And the software platform is implemented in Matlab7.4.0 (Mathworks Inc., Sherborn, MA). First, through linear and non-linear transformations, PET scans from all samples were spatially normalized into Montreal Neurological Institute brain space. Then, a Gaussian filter of 10 mm FWHM was used for smooth images over three-dimension space. After that, an automated anatomic labeling template was used to remove unrelated regions in PET images. Due to individual variation in FDG uptake, finally, each PET image was normalized to the range of 0 to 1 through following formula, where *v* is the voxel value of the image:

(12) $$\begin{array}{l} v_{normalization}=\;\frac{v-v_{min}}{v_{\max}-v_{min}} \end{array}$$

### Data Dimension Reduction

Locally linear embedding (LLE) (Roweis and Saul, 2000; Xin et al., 2005) was used to reduce the dimensionality of pre-processed PET data in all subjects, including the Huashan and Wuxi cohorts. Since high-dimensional features are often associated with many redundant and hidden important relationships, we need a more concise PET data representation. LLE is a typical manifold learning algorithm that has been used to reduce the dimensionality of medical images (Liu et al., 2013). LLE solves globally non-linear problems using locally linear fitting, which means a sample x1 can be represented linearly by several samples from its k neighborhoods:

(13) $$\begin{array}{l} {\text{x}}_{1}={\text{w}}_{12}\text{x}2+{\text{w}}_{13}{\text{x}}_{3}+\ldots +{\text{w}}_{1\text{k}}{\text{x}}_{\text{k}} \end{array}$$

Through LLE, we projected *x*_1_, *x*_2_, *x*_3_ … *x*_*k*_ onto a lower dimensional space $x_{1}^{\prime }$, $x_{2}^{\prime }$, $x_{3}^{\prime }$ … $x_{k}^{\prime }$ keeping the same linear relationship:

(14) $$\begin{array}{l} x_{1}^{\prime }\approx {\text{w}}_{\text{12}}{\text{x}}_{2}^{\prime }+{\text{w}}_{\text{13}}{\text{x}}_{3}^{\prime }+\ldots +{\text{w}}_{\text{1}k}{\text{x}}_{k}^{\prime } \end{array}$$

For high-dimensional data, LLE can maintain the local linear characteristics of the sample in the case of dimensionality reduction and map it to a low-dimensional global coordinate system, establishing a bridge between the high-dimensional data space and the low-dimensional latent space. In this paper, we use LLE to get a laconic representation of PET data. An automated method was used to optimize the LLE parameters number of neighbors, K, and corresponding dimensionality, D (Kayo, 2006).

### GLS-DBN for Feature Learning and Classification

Based on the proposed GLS-RBM model, we used three GLS-RBM stacks to form a sparse GLS-DBN network for feature relearning and classification of PD and NC samples. The input of the GLS-DBN model is the low-dimensional feature learned from original PET data using the LLE algorithm, and the output is the prediction result.

**Figure 2:**
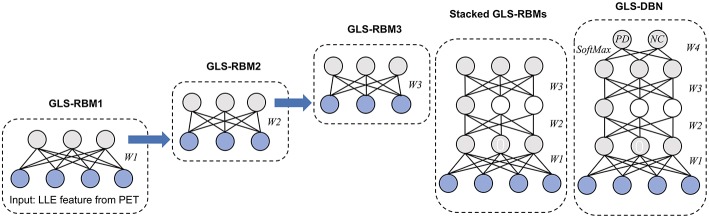
Structure of GLS-DBN for PD classification.

Figure 2 shows the structure of our GLS-DBN for PD classification. We used a greedy layer-wise algorithm for pre-training of the GLS-DBN. First, the weights (W1) were optimized to represent the distribution of the input data. Then the weights were frozen, the first level output was generated after input data through them. This output was used to train the next GLS-RBM, with training performed in the same way. Finally, on the top of the GLS-DBN, a SoftMax layer was added, all the layers performed supervised fine-tuning as one deep neural network.

In the training progress, all the training steps shared the same BP approach. The training set was randomly divided into several mini-batches or subsets, and the cost function was minimized using mini-batch gradient descent. At every iteration, only one mini-batch was used for minimization. After all the samples were used once for training, the training set was divided again so that batches in each echo had different samples. The initial learning rate was set to 0.0001.

The training progress of the model was carried out on the training & validation dataset from the Huashan cohort. Parameters were optimized through the mean accuracy of the validation dataset. Finally, the two test datasets (Test 1 and Test 2) were used to verify the performance of the trained model.

### Correlation Analyses

Before passing through the last layer of GLS-DBN, SoftMax function, the values of the two nodes indicate scores for PD and NC, respectively (Choi and Jin, 2018). The quantitative value of the PD node was defined as RiskScore, a score that indicates the proximity of PET data to PD or NC. In addition, RiskScore was correlated with Hoehn and Yahr scale (H&Y) and Unified Parkinson's Disease Rating Scale (UPDRS), in which Pearson correlation was used.

### Experimental Comparison

To verify the reliability of our algorithm, we compared it to traditional DBN and improved sparse DBN networks. The sparse representation capability in various improved models was examined, including Lee's model, based on a quadratic regularization term (Lee et al., 2007), Ji's model, based on rate distortion theory (Ji et al., 2014), Keyvanrad's model, based on normal distribution (Keyvanrad and Homayounpour, 2017), and Xu's model (Xu et al., 2018), based on Laplace distribution. In addition, we compared our model with traditional DBN (without sparse penalty), Cauchy distribution, and group lasso distribution to examine the effectiveness of overlapping group lasso model. All of these methods are based on different regularization term definitions.

In all experiments, the DBN-based algorithm used the same structure, namely the same layers and the same hidden units. Weights and biases were initialized to uniformly distributed random numbers.

We used the sparsity measurement method proposed by Hoyer to accurately calculate the sparsity of the feature representation learned by the model (Hoyer, 2004). The sparsity measurement method is as follows:

(15) $$\begin{array}{l} \text{sparseness}\left(x\right)=\;\frac{\sqrt{n}-\left(\sum_{i=1}^{n}|x_{i}|\right)/\sqrt{\sum_{i=1}^{n}x_{i}^{2}}}{\sqrt{n}-1} \end{array}$$

Where *x* is the input data, *n* is the dimension of input data, and the value range of sparsity is [0,1]. The closer to 1, the sparser x is. The sparsity of activation probability of hidden units in batch data was calculated first, and then the average sparsity of activation probability of all data hidden units was calculated.

Sensitivity, specificity and accuracy of the test datasets (Test 1 and Test 2) were used as indicators to measure the performance of the model. In order to further evaluate the performance of the proposed method, we used receiver operating characteristic (ROC) graph to visualize the result of contrast experiments. The area under the curve (AUC) of the ROC was also computed to quantitatively evaluate the classification performance.

In addition, to verify the robust of our proposed method, we conducted a second experiment analysis and reassigned the distribution of the training & validation set and test dataset according to the H&Y. Appendix A shows the demographic and clinical information on the second experiment analysis. Similar to the first experiment analysis, we repeated above steps to calculate sensitivity, specificity, accuracy and AUC of the training & validation and test datasets (Test 1 and Test 2).

## Results

### Determination of Parameters

Following an automated method (Kayo, 2006), the number of dimensions and nearest neighbors in LLE were set to 350 and 10.

To determine the optimal structure and parameters of the GLS-DBN model, including the scale parameter, location parameter, overlapping rate, and the number of hidden units, the greedy search algorithm was used in the whole training progress until the average accuracy of the validation dataset was optimized. These were chosen as the initial parameters for fine-tuning of the GLS-DBN.

Finally, hyper-parameters were set: number of hidden units, scale parameter, location parameter, and overlapping rate were set to 500, 1, 0.025, and 0.2. The maximum number of iterations of GLS-RBM and BP network were 50 and 300. Using features from LLE and GLS-DBN as classifiers, the classification experiment achieved 94% accuracy distinguishing PD and NC in the validation dataset.

### Classification Results

**Figure 3:**
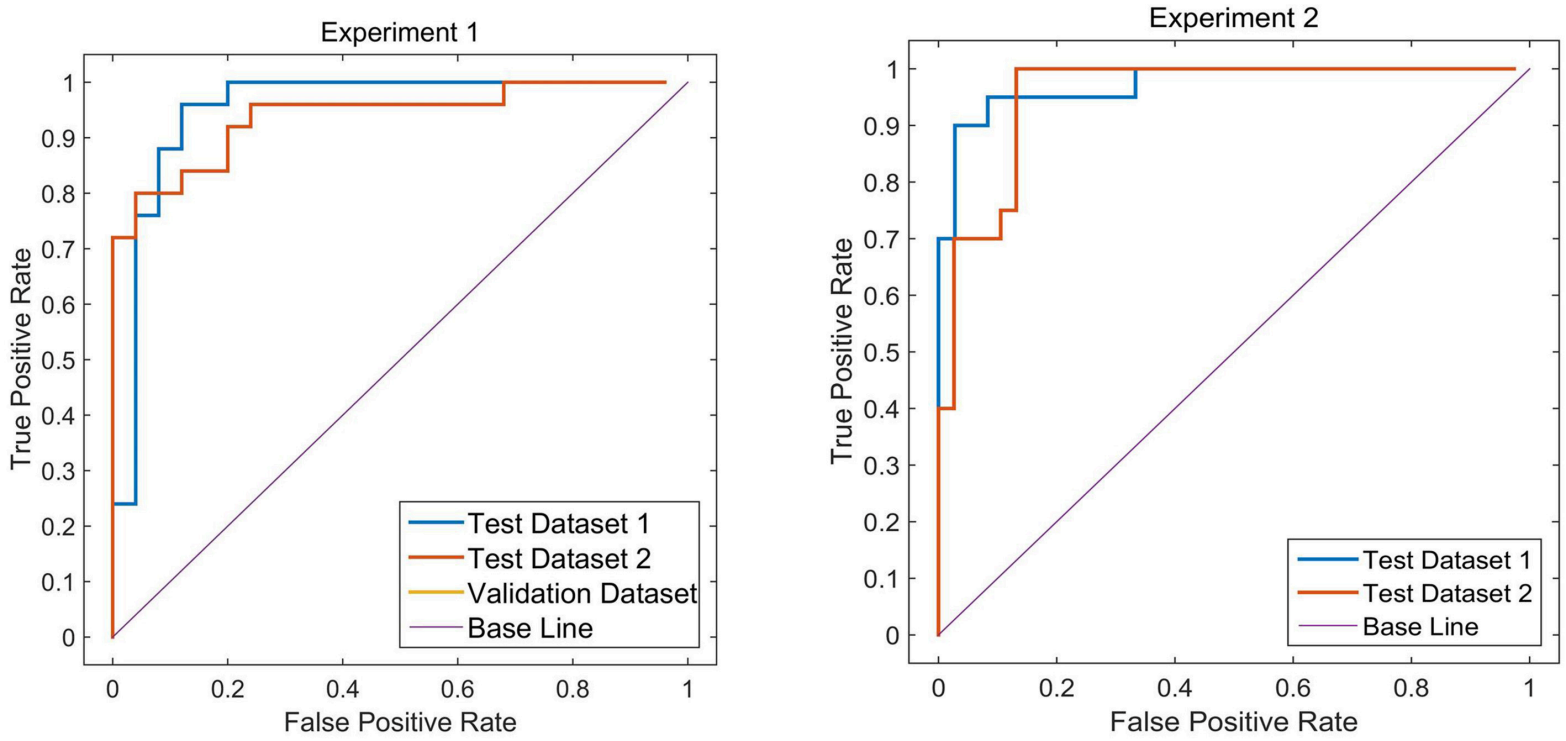
ROC curves on Validation dataset, Test dataset 1 and Test dataset 2.

Two different batches of data from the Huashan and Wuxi cohorts were used to validate the model's performance. Figure 3 and Table 2 show the final classification performance on the validation dataset, Test dataset 1, and Test dataset 2 under hyper-parameters in two experiments. Using features from LLE and GLS-DBN as classifiers, in Experiment 1, the classification experiment distinguishing PD and NC achieved 90.0% accuracy, 96% sensitivity, 84% specificity, and AUC of 0.9120 in Test dataset 1 and 86% accuracy, 92% sensitivity, 80% specificity, and AUC of 0.8992 in Test dataset 2; while in Experiment 2, the classification experiment distinguishing PD and NC achieved 88.0% accuracy, 92% sensitivity, 84% specificity, and AUC of 0.9320 in Test dataset 1 and 84% accuracy, 88% sensitivity, 80% specificity, and AUC of 0.8947 in Test dataset 2. As a result, we observed that the effect of different data distribution was slight for the classification. It means that our proposed model may have good robustness for other datasets.

**Table 2:** Classification performance on Validation and Test datasets.

		**Accuracy**	**Sensitivity**	**Specificity**	**AUC**
Experiment 1	Validation dataset	0.924	0.973	0.80	0.9325
	Test dataset 1	0.90	0.96	0.84	0.9120
	Test dataset 2	0.86	0.92	0.80	0.8992
Experiment 2	Validation dataset	0.9380	0.9642	0.834	0.9634
	Test dataset 1	0.88	0.92	0.84	0.9320
	Test dataset 2	0.84	0.88	0.80	0.8947

### Experimental Comparison

**Figure 4:**
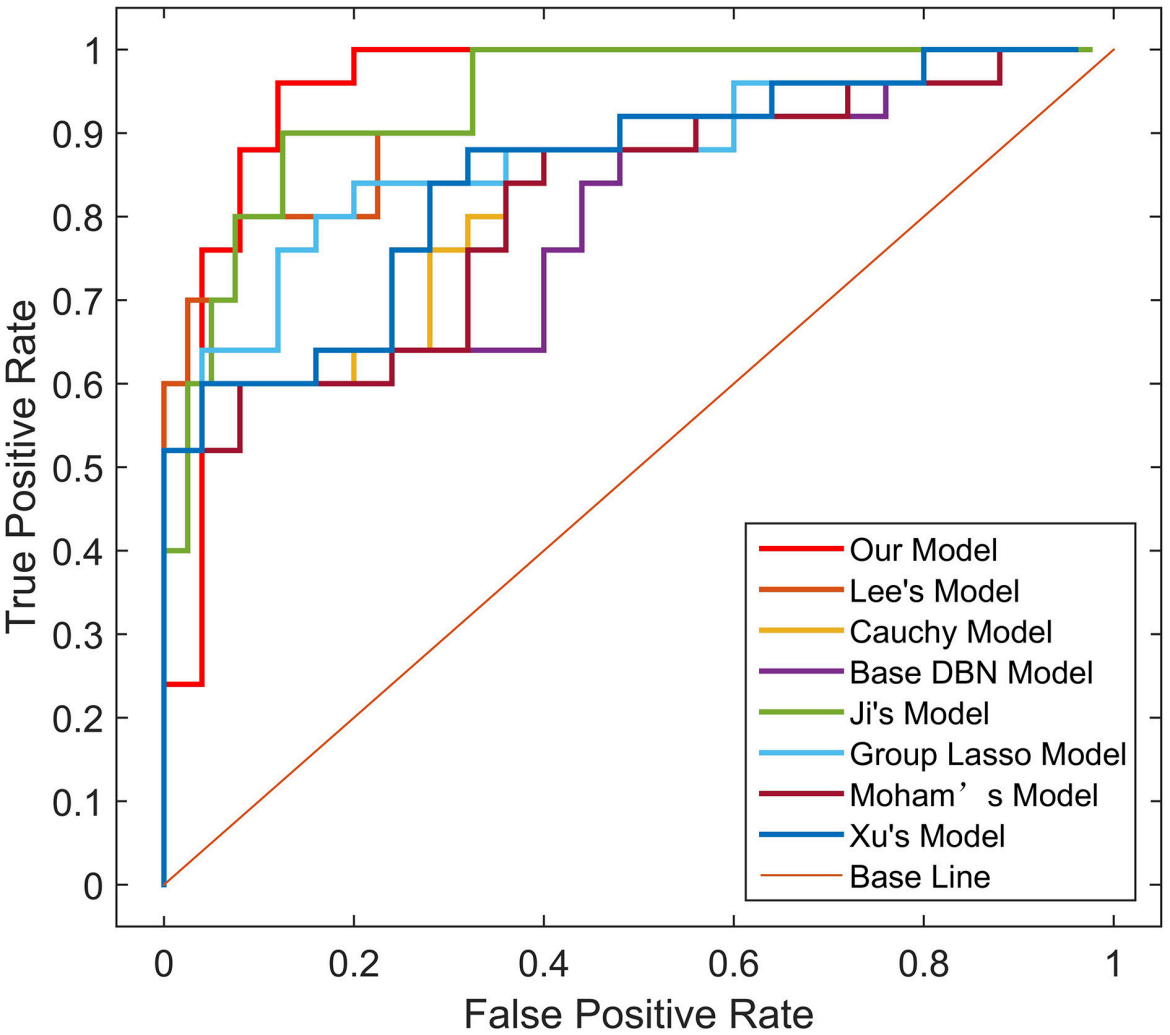
ROC curves on Test dataset 1 in different models.

**Figure 5:**
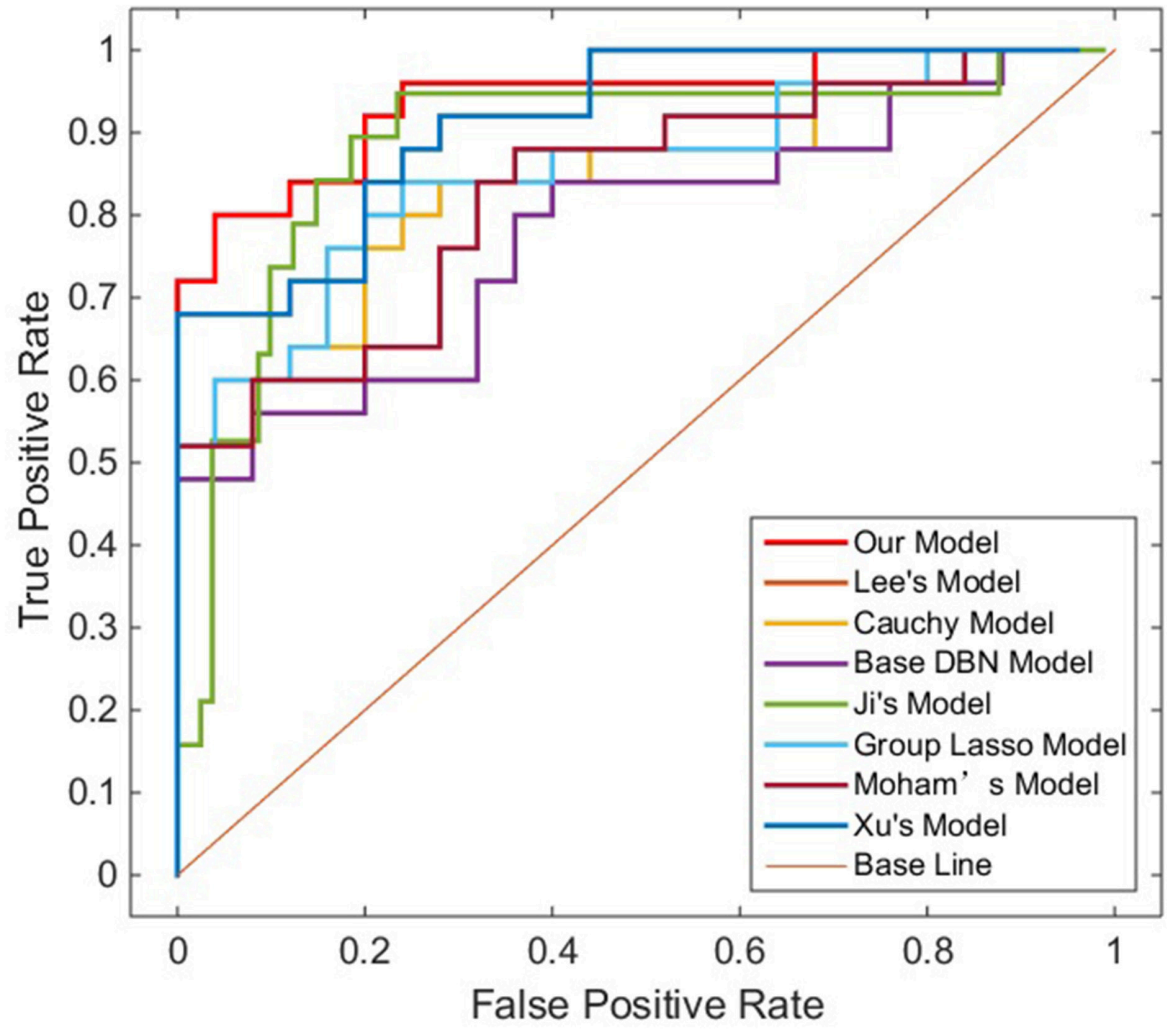
ROC curves on Test dataset 2 in different models.

To verify the recognition ability of the algorithm, the classification accuracies of different models were compared. The results are shown in Figures 4, 5 and Table 3.

**Table 3:** Classification performance on test datasets in different sparse models.

**Model**	**Dataset**	**Accuracy**	**Sensitivity**	**Specificity**	**AUC**
Base DBN model	Validation	0.7596	0.7845	0.6843	0.7769
	Test 1	0.66	0.72	0.60	0.7568
	Test 2	0.68	0.68	0.68	0.7408
Lee's model	Validation	0.9245	0.9353	0.8247	0.9275
	Test 1	0.88	0.925	0.70	0.91
	Test 2	0.84	**0.9630**	0.56	0.8726
Moham's model	Validation	0.7968	0.7164	0.6852	0.8087
	Test 1	0.70	0.72	0.68	0.7744
	Test 2	0.72	0.72	0.72	0.7904
Ji's model	Validation	0.8727	0.8867	0.7589	0.8977
	Test 1	0.86	**0.9750**	0.500	**0.9125**
	Test 2	0.84	0.96	0.45	0.8726
Xu's model	Validation	0.8443	0.8847	0.7964	0.8876
	Test 1	0.74	0.72	0.76	0.8096
	Test 2	0.80	0.80	0.80	0.8752
Cauchy model	Validation	0.8034	0.8181	0.7443	0.8232
	Test 1	0.72	0.72	0.72	0.7824
	Test 2	0.76	0.72	0.80	0.8016
Group Lasso model	Validation	0.898	0.9341	0.7877	0.9012
	Test 1	0.80	0.72	**0.88**	0.8320
	Test 2	0.78	0.72	**0.84**	0.8160
Our model	Validation	0.935	0.925	0.85	0.9487
	Test 1	**0.90**	0.96	0.84	0.9120
	Test 2	**0.86**	0.92	0.80	**0.8992**

*The bold values represents means the highest value of accuracy, sensitivity, specificity and AUC on test dataset 1 and test dataset 2 in different models*.

As shown in Table 3, Figures 4, 5, GLS-DBN achieved the best accuracy on the training and test datasets when using the same network structure. GLS-DBN resulted in improved performance when compared to the traditional DBN classifier. Although the best sensitivities in the test dataset were seen in DBN models based on quadratic regularization and rate distortion, the specificities of these models were quite low (0.56 and 0.50) indicating that the rate of missed diagnosis is low but the rate of misdiagnosis is high. Improved DBN model-based group lasso achieved the best sensitivity, but the specificity was relatively low. These results indicate that our model has the ability to balance specificity and sensitivity when the best accuracy is reached. The accuracy in the Test 2 dataset was not significantly lower than the accuracy in the Test 1 dataset, demonstrating the generalizability of our model. Our model also achieved the best AUC in the Test 2 dataset and close to the optimal result in the Test 1 dataset. In a comprehensive sense, compared with other sparse DBN models based on different sparse penalties, our model optimizes the prediction of PD diagnosis.

### Correlation Analyses

**Figure 6:**
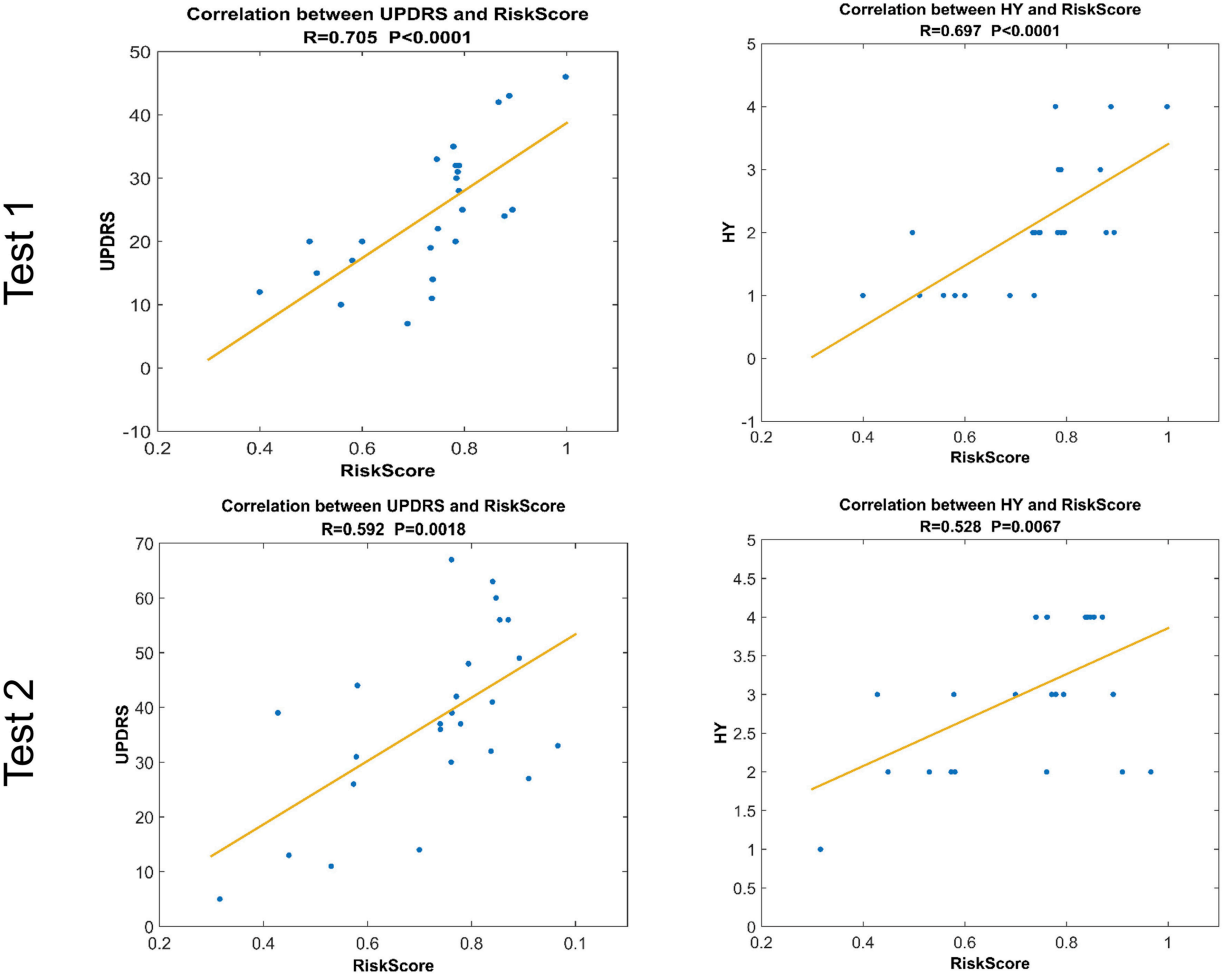
Correlation between output of the network and clinical scale value.The last layer provides an output score for NC or PD converter, defined as RiskScore.

RiskScore calculated from the GLS-DBN model was significantly correlated with clinical scale value in the Test dataset, shown in Figure 6. RiskScore was significantly positively correlated with UPDRS (*r* = 0.705, *P* < 0.0001), and HY (*r* = 0.697, *p* < 0.0001) in Test 1, UPDRS (*r* = 0.592, *P* = 0.0018), and HY (*r* = 0.528, *p* = 0.0067) in Test 2.

These results show that RiskScore could be used as a quantitative biomarker for early diagnosis of Parkinson's disease.

## Discussion

In this paper, we used an improved sparse DBN named GLS-DBN for the diagnosis of PD. Compared with other sparse DBN models based on different sparse penalties, our model showed better performance in classification of PD and NC, demonstrating that GLS-DBN can be used for effectively learning superior feature representation from small neuroimaging data. Results from other datasets also proved the preferable generalizability of GLS-DBN.

In addition, we compared the results of other similar studies to our results. Due to differences in datasets, number of samples, and methods of feature selection and reduction, while the accuracy was not the best, our proposed method still exceeded most relevant studies. Further, the sensitivity of our result was significantly higher than those automated classifications (96 vs. 86.67%, 96 vs. 84.4%) (Fung and Stoeckel, 2007; Rana et al., 2015). And the accuracy is closer to those methods based on voxel statistical analyses (90 vs. 90.9%, 90 vs. 86.5%) (Eckert et al., 2005). Our results also show a better specificity compared with automated classifications. Our automated method based deep learning performs better than traditional CAD methods and approaches results with manual diagnosis.

In terms of feature extraction and dimension reduction, the research of Rana et al. considered five brain areas, while the features used in our experiment were chosen from the whole brain (Rana et al., 2015). For high-dimensional PET data, LLE has learned the potential non-linear expression in PET image data and embedded the features after dimensionality reduction into linear coordinates, which can map the PET data of PD and NC to different feature spaces, that is, subjects with different clinical manifestations have different distribution of brain features (Roweis and Saul, 2000). So, LLE reduces the recognition error caused by redundant information and significantly improves the feature difference between PD and NC samples, which is crucial for later feature learning and classification.

**Figure 7:**
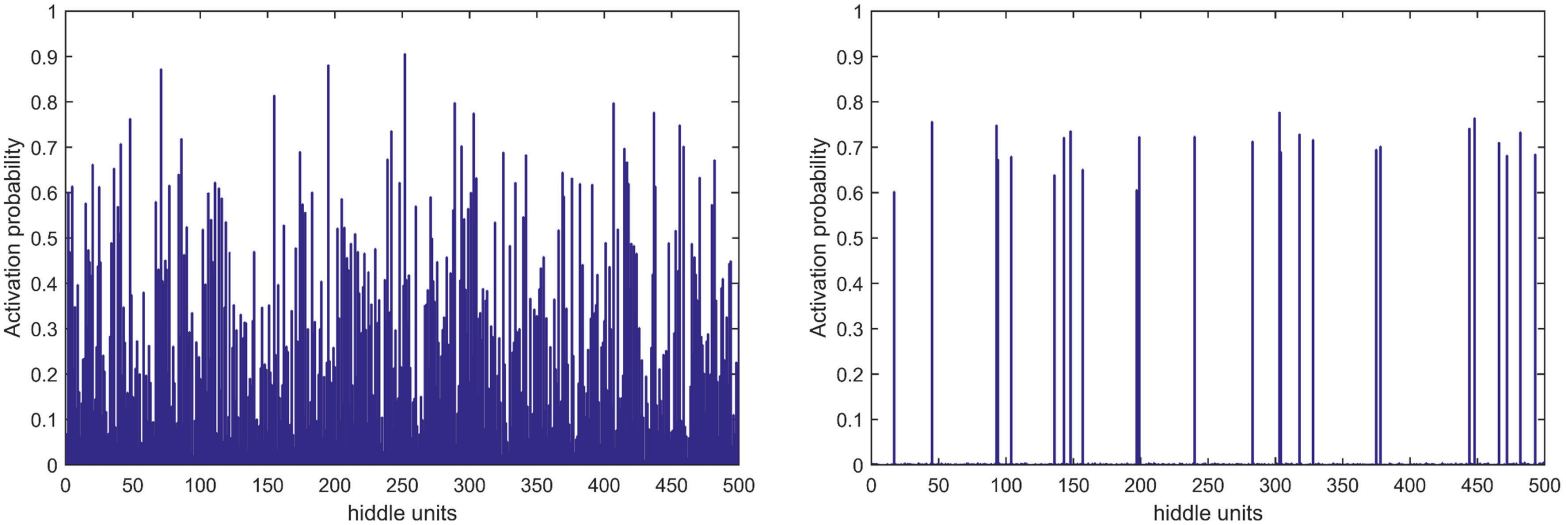
Activation probability of hidden units obtained by RBM (left) and GLS-RBM (right).

In terms of feature learning, almost all studies using pattern recognition in PD diagnosis use features directly for classification, without the progress of feature relearning. In this study, the GLS-DBN model based on a deep learning algorithm can re-encode features before classification, which improves accuracy. The experimental results also show that GLS-DBN can learn more appropriate features in PET data compared with the traditional RBM and DBN algorithms. One possible reason is that sparse coding learns helpful low-dimensional feature representations from unlabeled data. By constraining the hidden layer, GLS-RBM can obtain a simpler and more structured weight pattern, thus avoiding the redundant and sequential-value code that RBM may produce that RBM may produce. Figure 7 shows the activation probability of hidden units caused by an input data, in other words, the representation of this input data obtained by traditional RBM and GLS-RBM. As we can see from Figure 7, the activation probability of RBM approaching is much lower than that of GLS-RBM, while the activation probability of the hidden units in sparse-RBM is very close to 0, which means DBN-based sparse penalty can learn a sparser representation of input data. Table 4 shows the results of sparseness in different improved DBN models. Although Lee's model and Ji's model achieved the best sparseness (0.8594, 0.8239), classification performance in Table 3 showed high sensitivity (0.925, 0.9750 in Test 1, 0.9630, 0.96 in Test 2) but low specificity (0.70, 0.56 in Test 1, 0.50, 0.45 in Test 2). Compared with other sparse models, our model achieved the highest sparseness (0.8011 vs. 0.6619, 0.7145, 0.7473, 0.7963, 0.7852) and also the best classification performance. The results in Table 4 show that the sparsest DBN model does not represent the most suitable model for learning useful low-level feature representation of PET image patterns. Combining the results of Table 3, through the adjustment of parameters, the GLS-DBN base overlapping group lasso model can achieve optimal sparseness, and balance specificity and sensitivity while ensuring the accuracy of the model.

**Table 4:** Average sparseness of different models.

**Model**	**Base DBN model**	**Lee's model**	**Ji's model**	**Xu's model**	**Cauchy model**	**Keyvanrad's model**	**Group Lasso model**	**Our model**
Sparseness	0.6619	0.8594	0.8239	0.7145	0.7473	0.7963	0.7852	0.8011

PD reflects in a small part of the brain pathology, causing differences in only part of the brain compared to healthy people. When using LLE dimensionality reduction for 3D PET images, there may be a group relationship between features; moreover, it is possible that there is overlap of features between groups. The overlapping group lasso model takes this relationship into account, and using a sparse penalty term effectively suppresses expression of some redundant features. This increases the feature difference between PD and NC samples, achieving improved classification. The results of Test 1 from the Huashan cohort show the excellent performance of our model. The results of Test 2 from the Wuxi cohort show the reliability of the model, and also its generalizability to other datasets.

**Figure 8:**
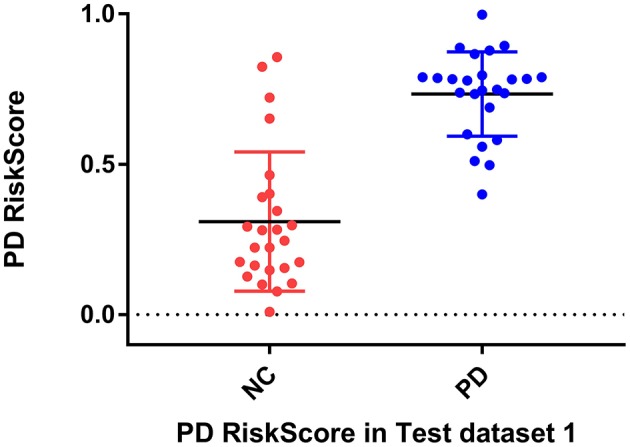
RiskScore of PD and NC.

**Figure 9:**
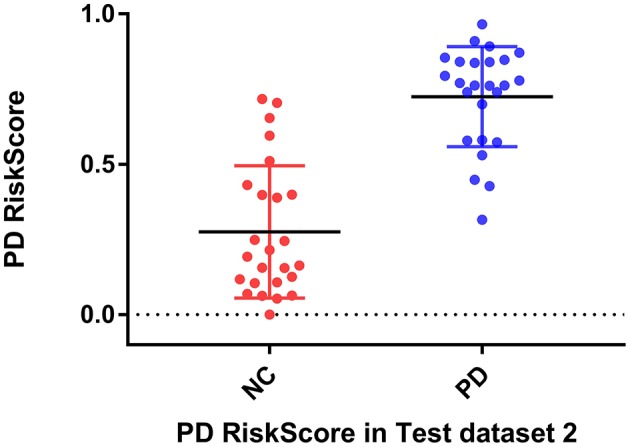
RiskScore of PD and NC in test dataset 1 and in test dataset 2.

Considering the clinical effectiveness of the results, including HY and UPDRS, this paper analyzed the correlation between RiskScore and the clinical scale, shown in Figure 6. RiskScore calculated from the GLS-DBN model was significantly correlated with clinical scale value (0.705, 0.697, *p* < 0.001; 0.592, 0.528, *p* < 0.01) in the Test dataset, indicating that the features learned by GLS-DBN correlate with clinical information. To better describe the discriminability of the results, we conducted a statistical analysis of the risk values, and the distribution of RiskScore in PD and NC are shown in Figures 8, 9. RiskScore of PD was significantly higher than that of NC (0.31 ± 0.23 and 0.73 ± 0.14, *p* < 0.01 in Test 1, 0.26 ± 0.22 and 0.72 ± 0.17, *p* < 0.01 in Test 2). These results indicate that this method can effectively classify PD and NC, and that RiskScore can be used as a quantitative biomarker for early diagnosis of PD.

## Limitations

Despite the impressive performance of the proposed method, the method also has some limitations and disadvantages. First, trial and error was used to determine the learning rate, and the parameter values in the network structure were optimized through a large number of experiments, causing a relatively large time complexity of the algorithm. Proper selection of parameters merits further studies; we propose using optimization algorithms such as a grid search algorithm to search for the optimal parameter combination as a next step. Second, while our work has a large number of subjects compared with several recent studies, it's still not enough to generalize our experimental results. Further, our work focuses on PET features only. Multimodal data, such as MRI and diffusion tensor imaging features, can be used for feature confusion and classification. Third, our classifier is a BP network, and other classifiers such as extreme learning machine and support vector machine can be combined with DBN. Combining other classifiers with DBN would allow the last layer before BP to be features learned from the DBN model and as an input for traditional classifiers, possibly improving classification results. Finally, as our study is based on PD and NC samples, it would be meaningful and of vital importance to further grade of different forms of Parkinson's disease or distinguish idiopathic Parkinson's disease from other forms of degenerative Parkinson's disease.

## Conclusion

In this paper, we introduced a sparse feature learning framework in PD early diagnosis. GLS-DBN model accurately classifies patients into diagnostic groups with limited image processing and provides a quantitative biomarker which can predict early Parkinson's disease. Longitudinal changes of rating scales about movement disorders (UPSRS and H&Y), was significantly correlated with the output value of prediction model. In the future, our approach may be used in independent cohorts, and as an accurate biomarker, it could identify appropriate prodromal patients who might benefit from early intervention.

## Ethics Statement

This study was carried out in accordance with the recommendations of Ethics committee of longhua hospital affiliated to University of Traditional Chinese Medicine with written informed consent from all subjects. All subjects gave written informed consent in accordance with the Declaration of Helsinki. The protocol was approved by the longhua hospital affiliated to University of Traditional Chinese Medicine.

## Author Contributions

All authors listed have made a substantial, direct and intellectual contribution to the work, and approved it for publication.

### Conflict of Interest Statement

The authors declare that the research was conducted in the absence of any commercial or financial relationships that could be construed as a potential conflict of interest.

## References

[B1] Brajkovic L. Kostic V. Sobicsaranovic D. Stefanova E. Jecmenicalukic M. Jesic A.. (2017). The utility of FDG-PET in the differential diagnosis of Parkinsonism. Neurol. Res. 39, 675–684. 10.1080/01616412.2017.1312211 28378615

[B2] Brosch T. Yoo Y. Li D. K. B. Traboulsee A. Tam R. (2014). Modeling the variability in brain morphology and lesion distribution in multiple sclerosis by deep learning. Med Image Comput Comput. Assist Interv. 17(Pt 2):462–9. 10.1007/978-3-319-10470-6_58 25485412

[B3] Chandra B. Sharma R. K. (2016). Fast learning in deep neural networks. Neurocomputing 171, 1205–1215. 10.1016/j.neucom.2015.07.093

[B4] Chen Y. Lin Z. Xing Z. Gang W. Gu Y. (2017). Deep learning-based classification of hyperspectral data. IEEE J. Select. Top. Appl. Earth Observ. Remote Sens. 7, 2094–2107. 10.1109/JSTARS.2014.2329330

[B5] Choi H. Jin K. H. (2018). Predicting cognitive decline with deep learning of brain metabolism and amyloid imaging. Behav. Brain Res. 344, 103–109. 10.1016/j.bbr.2018.02.017 29454006

[B6] Dabrowska M. Schinwelski M. Sitek E. J. Muraszko-Klaudel A. Brockhuis B. Jamrozik Z.. (2015). The role of neuroimaging in the diagnosis of the atypical parkinsonian syndromes in clinical practice. Neurol. Neurochir. Pol. 49, 421–431. 10.1016/j.pjnns.2015.10.002 26652877

[B7] Eckert T. Barnes A. Dhawan V. Frucht S. Gordon M. F. Feigin A. S.. (2005). FDG PET in the differential diagnosis of parkinsonian disorders. Neuroimage 26, 912–921. 10.1016/j.neuroimage.2005.03.012 15955501

[B8] Fischer A. Igel C. (2012). An introduction to restricted Boltzmann machines, in Iberoamerican Congress on Pattern Recognition (Buenos Aires). 10.1007/978-3-642-33275-3_2

[B9] Fung G. Stoeckel J. (2007). SVM feature selection for classification of SPECT images of Alzheimer's disease using spatial information. Knowled. Inform. Syst. 11, 243–258. 10.1007/s10115-006-0043-5

[B10] Garraux G. Phillips C. Schrouff J. Kreisler A. Lemaire C. Degueldre C. (2013). Multiclass classification of FDG PET scans for the distinction between Parkinson's disease and atypical parkinsonian syndromes ⋆⋆. Neuroimage Clin. 2, 883–893. 10.1016/j.nicl.2013.06.004 24179839 PMC3778264

[B11] Halkias X. Paris S. Glotin H. (2013). Sparse penalty in deep belief networks: using the mixed norm constraint. Comput Sci. Available online at: http://arxiv.org/abs/1301.3533

[B12] Hinton G. E. Osindero S. Teh Y. W. (2006). A fast learning algorithm for deep belief nets. Neural Comput. 18, 1527–1554. 10.1162/neco.2006.18.7.1527 16764513

[B13] Hoyer P. O. (2004). Non-negative matrix factorization with sparseness constraints. J. Mach. Learn. Res. 5, 1457–1469. 10.1016/j.neucom.2011.09.024

[B14] Ji N. N. Zhang J. S. Zhang C. X. (2014). A sparse-response deep belief network based on rate distortion theory. Pattern Recognit. 47, 3179–3191. 10.1016/j.patcog.2014.03.025

[B15] Jian W. Cai Q. Chang Q. Zurada J. M. (2017). Convergence analyses on sparse feedforward neural networks via group lasso regularization ⋆. Inform. Sci. Int. J. 381, 250–269. 10.1016/j.ins.2016.11.020

[B16] Juh R. Kim J. Moon D. Choe B. Suh T. (2004). Different metabolic patterns analysis of Parkinsonism on the 18F-FDG PET. Eur. J. Radiol. 51, 223–233. 10.1016/S0720-048X(03)00214-6 15294329

[B17] Kayo O. (2006). Locally linear embedding algorithm – Extensions and applications. Value Eng. Available online at: http://jultika.oulu.fi/Record/isbn951-42-8041-5

[B18] Keyvanrad M. A. Homayounpour M. M. (2017). Effective sparsity control in deep belief networks using normal regularization term. Knowl. Inf. Syst. 53, 533–550. 10.1007/s10115-017-1049-x

[B19] Lee H. Ekanadham C. Ng A. Y. (2007). Sparse deep belief net model for visual area V2, in International Conference on Neural Information Processing Systems (Vancouver, BC). 10.1.1.87.2404&rep=rep1&type=pdf

[B20] Liu X. Peng C. Yang J. Zhao D. Zaiane O. (2017). “Group guided sparse group lasso multi-task learning for cognitive performance prediction of Alzheimer?s disease,” in International Conference on Brain Informatics (Beijing). 10.1007/978-3-319-70772-3_19

[B21] Liu X. Tosun D. Weiner M. W. Schuff N. Alzheimer's Disease Neuroimaging I. (2013). Locally linear embedding (LLE) for MRI based Alzheimer's disease classification. Neuroimage 83, 148–157. 10.1016/j.neuroimage.2013.06.033 23792982 PMC3815961

[B22] Liu Y. Zhou S. Chen Q. (2011). Discriminative deep belief networks for visual data classification. Pattern Recognit. 44, 2287–2296. 10.1016/j.patcog.2010.12.012

[B23] Lü R. Guan X. Li X. Hwang I. (2016). A large-scale flight multi-objective assignment approach based on multi-island parallel evolution algorithm with cooperative coevolutionary. Sci. China Inform. Sci. 59:072201 10.1007/s11432-015-5495-3

[B24] Luo H. Shen R. Niu C. (2010). Sparse group restricted boltzmann machines*. Statistics*. Available online at: https://arxiv.org/pdf/1008.4988.pdf

[B25] Matthews D. C. Hedva L. Ana L. Andrews R. D. Anat M. Wernick M. N.. (2018). FDG PET Parkinson's disease-related pattern as a biomarker for clinical trials in early stage disease. NeuroImage Clin. 20, 572–579. 10.1016/j.nicl.2018.08.006 30186761 PMC6120603

[B26] Mei X. Yong M. Fan F. Chang L. Liu C. Huang J. (2015). Infrared ultraspectral signature classification based on a restricted Boltzmann machine with sparse and prior constraints. Int. J. Remote Sens. 36, 4724–4747. 10.1080/01431161.2015.1079664

[B27] Meles S. K. Teune L. K. De Jong B. M. Dierckx R. A. Leenders K. L. (2017). Metabolic imaging in parkinson disease. J. Nucl. Med. Off. Public. Soc. Nuclear Med. 58, 23–28. 10.2967/jnumed.116.183152 27879372

[B28] Meyer P. T. Frings L. Rucker G. Hellwig S. (2017). (18)F-FDG PET in parkinsonism: differential diagnosis and evaluation of cognitive impairment. J. Nucl. Med. 58, 1888–1898. 10.2967/jnumed.116.186403 28912150

[B29] Politis M. Pagano G. Niccolini F. (2017). Imaging in Parkinson's Disease. Int. Rev. Neurobiol. 132:233–274. 10.1016/bs.irn.2017.02.015 28554409

[B30] Postuma R. B. Berg D. (2017). The new diagnostic criteria for parkinson's disease. Int. Rev. Neurobiol. 132, 55–78. 10.1016/bs.irn.2017.01.008 28554421

[B31] Prasetio M. D. Hayashida T. Nishizaki I. Sekizaki S. (2018). Deep belief network optimization in speech recognition, in International Conference on Sustainable Information Engineering and Technology (Malang). 10.1109/SIET.2017.8304124

[B32] Rana B. Juneja A. Saxena M. Gudwani S. Senthil Kumaran S. Agrawal R. K. (2015). Regions-of-interest based automated diagnosis of Parkinson's disease using T1-weighted MRI. Expert Syst. Appl. 42, 4506–4516. 10.1016/j.eswa.2015.01.062

[B33] Ranzato M. Boureau Y. L. Lecun Y. (2007). Sparse feature learning for deep belief networks, in International Conference on Neural Information Processing Systems (Vancouver, BC). Available online at: https://dl.acm.org/citation.cfm?id=2981672

[B34] Rao N. S. Nowak R. Cox C. Rogers T. (2015). Classification with the Sparse Group Lasso. IEEE Transac. Signal Process. 64, 448–463. 10.1109/TSP.2015.2488586

[B35] Roweis S. T. Saul L. K. (2000). Nonlinear dimensionality reduction by locally linear embedding. Science 290, 2323–2326. 10.1126/science.290.5500.2323 11125150

[B36] Rui X. Yang L. (2017). A multi-task learning framework for emotion recognition using 2D continuous space. IEEE Transact. Affect. Comput. 8, 3–14. 10.1109/TAFFC.2015.2512598

[B37] Siqi L. Sidong L. Weidong C. Hangyu C. Sonia P. Ron K. (2015). Multimodal neuroimaging feature learning for multiclass diagnosis of Alzheimer's disease. IEEE Transac. Biomed. Eng. 62, 1132–1140. 10.1109/TBME.2014.2372011 PMC4394860 25423647

[B38] Suk H. I. Lee S. W. Shen D. Initiative T. (2015). Latent feature representation with stacked auto-encoder for AD/MCI diagnosis. Brain Struct. Func. 220, 841–859. 10.1007/s00429-013-0687-3 24363140 PMC4065852

[B39] Tang C. C. Poston K. L. Eckert T. Feigin A. (2010). Differential diagnosis of parkinsonism: a metabolic imaging study using pattern analysis. Lancet Neurol. 9, 130–131. 10.1016/S1474-4422(10)70002-8 20061183 PMC4617666

[B40] Tripathi M. Tang C. C. Feigin A. Lucia I. D. Nazem A. Dhawan V.. (2015). Automated differential diagnosis of early parkinsonism using metabolic brain networks: a validation study. J. Nuclear Med. 57, 60–66. 10.2967/jnumed.115.161992 26449840

[B41] Xin G. De-Chuan Z. Zhi-Hua Z. (2005). Supervised nonlinear dimensionality reduction for visualization and classification. IEEE Transac. Syst. Man Cybernet. Part B Cybernet. Publicat. IEEE Syst. Man Cybernet. Soc. 35, 1098–1107. 10.1109/TSMCB.2005.850151 16366237

[B42] Xu Y. Li B. B. Song W. (2018). Research on improved deep belief network classification algorithm. J. Front. Comput. Sci. Technol. 13, 596–607. 10.3778/j.issn.1673-9418.1804002

[B43] Yoshida Y. Miyato T. (2017). Spectral norm regularization for improving the generalizability of deep learning. Available online at: https://arxiv.org/pdf/1705.10941.pdf

[B44] Yuan L. Yu L. Yi Z. Yue C. (2018). Speech bottleneck feature extraction method based on overlapping group lasso sparse deep neural network. Speech Commun. 99, 56–61. 10.1016/j.specom.2018.02.005

[B45] Zheng W. L. Lu B. L. (2017). Investigating critical frequency bands and channels for EEG-based emotion recognition with deep neural networks. IEEE Trans. Auton. Ment. Dev. 7, 162–175. 10.1109/TAMD.2015.2431497

