# Supplementary material for: Use of Overlapping Group LASSO Sparse Deep Belief Network to Discriminate Parkinson's Disease and Normal Control
Source: Front Neurosci. 2019 Apr 29;13:396. doi: 10.3389/fnins.2019.00396 (PMC6501727; doi:10.3389/fnins.2019.00396)
Supplement: Supplementary file 1 [file Data_Sheet_1.docx]

Appendix A: Demographic and clinical information for the second experiment analysis

|  | **Cohort** |  | **N** | **Gender(M/F)** | **Age(years)** | **H&Y** | **UPDRS** |
| --- | --- | --- | --- | --- | --- | --- | --- |
| **Huashan Hospital Cohort** | **Training & Validation dataset** | **NC** | 175 | 96/79 | 50.5(25) | N/A | N/A |
|  |  | **PD** | 75 | 50/25 | 55.5(17.5) | 2(1) | 23(18.4) |
|  |  | **P-Value** | - | 0.5846^a^ | 0.648^b^ | - | - |
|  | **Test dataset (Test 1)** | **NC** | 25 | 12/13 | 49.5(24.5) | N/A | N/A |
|  |  | **PD** | 25 | 15/10 | 54.5(15.5) | 2 (1) | 24(17.8) |
|  |  | **P-Value** | - | 0.8847^a^ | 0.3658^b^ | - | - |
| **Wuxi 904 Hospital Cohort** | **Test dataset (Test 2)** | **NC** | 25 | 12/13 | 59(9) | N/A | N/A |
|  |  | **PD** | 25 | 19/6 | 65(11.5) | 2.5(1.5) | 28(18.5) |
|  |  | **P-Value** | - | 0.1884^a^ | 0.771^b^ | - | - |
